# Dynamic Changes of Arc Expression in Dorsal Striatum of Mice After Self-Administration of Sucrose

**DOI:** 10.3389/fncel.2021.654521

**Published:** 2021-05-19

**Authors:** Xue Li, Jing-Wang Zhao, Qian Ding, Cheng Wu, Wan-Qi Li, Yan-Chen Guo, Di Wang, Guang-Qing Xu, Ti-Fei Yuan, Wan-Kun Gong, Yue Lan

**Affiliations:** ^1^Department of Rehabilitation Medicine, Guangzhou First People’s Hospital, Guangzhou Medical University, Guangzhou, China; ^2^School of Rehabilitation Science, Shanghai University of Traditional Chinese Medicine, Shanghai, China; ^3^Department of Rehabilitation Medicine, Guangzhou First People’s Hospital, School of Medicine, South China University of Technology, Guangzhou, China; ^4^Shanghai Key Laboratory of Psychotic Disorders, Shanghai Mental Health Center, Shanghai Jiao Tong University School of Medicine, Shanghai, China; ^5^Department of Rehabilitation Medicine, Guangdong Provincial People’s Hospital, Guangdong Academy of Medical Sciences, Guangzhou, China; ^6^Translational Research Institute of Brain and Brain-Like Intelligence, Shanghai Fourth People’s Hospital Affiliated to Tongji University School of Medicine, Shanghai, China; ^7^Co-innovation Center of Neuroregeneration, Natong University, Nantong, China

**Keywords:** Arc, the dorsomedial striatum, the dorsolateral striatum, instrumental learning, self-administration

## Abstract

Region-specific plasticity in the striatal circuit plays an important role in the development and long-term maintenance of skills and sequential movement procedures. Studies investigating the molecular substrates that contribute to the plasticity changes during motor skill processes have documented a transition in expression from the dorsomedial striatum (DMS) to the dorsolateral striatum (DLS); however, few studies have explored the expression pattern of molecular substrates in the dorsal striatum during progression of instrumental learning. To address this issue, the activity-regulated cytoskeleton-associated protein (Arc) expressions in the subregional dorsal striatum were analyzed during the early and late learning phases of the 10-day sucrose self-administration process. We found that Arc protein is primarily detected in the DMS only in the initial learning stage; however, it is expressed in the DLS during both early and late learning stages. Moreover, Arc expression in the DMS correlated with the number of rewards received later in the training. These data indicated that the Arc expression in subregions of the dorsal striatum shows region-specific transfer and that Arc expression in the DMS contributes to obtaining reward in later learning stage during the process of instrumental learning.

## Introduction

Instrumental or operant conditioning can be considered as learning about specific behavior and its consequences. Such processes are believed to be flexible and convertible, to produce rewarding outcomes or to avoid undesirable outcomes (Corbit, 2018). One of the most commonly used tasks to investigate instrumental conditioning is the self-administration (SA) paradigm, wherein hungry or thirsty animals perform seemingly random movements, such as nose-poking, to obtain food or water. Previous studies have shown that chronic access to highly palatable foods or sucrose water can promote a shift from goal-directed performance to habit-based performance (Avena et al., 2008; Kenny, 2011; Furlong et al., 2014). Several studies have investigated the neuronal activity during this shift process; however, the neural plasticity mechanism controlling instrumental learning is not well characterized.

The basal ganglia were earlier believed to control executive motor functions; however, several studies have demonstrated that the basal ganglia also affect cognition and motivational behavior (Graybiel, 2008; Witt, 2021). As a major part of the basal ganglia, the dorsal striatum plays an essential role in the acquisition of new skills. The striatum is composed of two parts, i.e., the dorsal medial striatum (DMS) and the dorsal lateral striatum (DLS). The DMS receives afferents from the prefrontal and associative cortex, and mainly participates in goal-oriented learning (Balleine and O’Doherty, 2010). The DLS receives afferents from the sensorimotor cortex (Voorn et al., 2004; Hawes et al., 2015) and is mainly involved in habitual learning. Numerous studies, including our previous study (Gong et al., 2020), have documented a transition in the engaged dorsal striatal subregions (from dorsomedial to dorsolateral) with the shift in skill performance from an initial attentive phase to a more automatic or habitual phase during motor skill tasks (Yin et al., 2008; Balleine and O’Doherty, 2010; Kupferschmidt et al., 2017; Bergstrom et al., 2018). In a long SA test, the DLS was found necessary for habitual performance. However, as a more complex behavioral paradigm than skill learning, the pattern of involvement of the two major sub-regions of the dorsal striatum in the SA learning process has rarely been explored.

Activity-regulated cytoskeleton-associated protein (Arc) is an immediate early gene that has been strongly suggested as a molecular marker for neuronal plastic changes underlying the formation and stabilization of long-term memory in striatum, hippocampus, and cortex (Noe et al., 2019; Guan et al., 2021). Several studies have documented an increase in the expression of Arc in the dorsal and ventral striatum after cocaine SA (Gao et al., 2017). However, the expression pattern in the dorsal striatum in different stages of instrumental learning has not been explored yet. In this study, we attempted to determine the regional Arc expression in the different regions of the dorsal striatum during formation and consolidation of instrumental learning. The sucrose SA task was used to assess the acquisition and consolidation of instrumental learning. The Arc-positive cells in the DMS and DLS were calculated during the early and later phases in the 10-day sucrose SA process. Furthermore, by assessing the correlation between density of Arc-positive cells in the dorsal striatum and behavior, we investigated the link between dorsal striatal Arc expression and instrumental learning in mice. Our results suggested that the Arc expression in subregions of dorsal striatum shows region-specific transfer and that Arc expression in the DMS contributes to obtaining a reward in the later learning stage, during the process of instrumental learning.

## Materials and Methods

### Animals

Male C57BL/6J mice (weight: 25–30 g) were purchased from the Shanghai Reagan Biotechnology Co., Ltd. Mice were housed in standardized environmental conditions (12 h light, 50% humidity, temperature: 18–22°C) and provided *ad libitum* access to water and food. Male mice aged 6 to 8 weeks were used for behavioral experiments. All animal experiments were approved by the Commission on animal experiments and application of the Shanghai Jiao Tong University School of Medicine. All efforts were made to minimize the pain caused to animals. Only the minimum number of animals required to generate credible data were used in this study.

### Sucrose Self-Administration

C57 mice were handled by the experimenter for 3 min each day, starting at least 4 days before the sucrose SA training, in an operant conditioning box (Anilab, China). The program was set as follows: An active nose-poke led to delivery of sucrose (10% solution; 0.083 mL/infusion; duration: 5 s) followed by a tone (20 dB) for 5 s + turning off of house lights at each sucrose delivery. There was a 20 s interval after each infusion. During this period, only the number of nose pokes was recorded, but there was no sucrose delivery.

Prior to the initiation of the experiment, the mice were deprived of water and food for 12 h. The training lasted 3 h (from 8:00 to 11:00) and was carried out in a soundproof and ventilated operating cage. Mice were trained to respond to a 10% sucrose solution at a fixed ratio of 1 (FR1). Each active poking of the nose delivered 0.083 mL of sucrose solution. After 10 days of training, the number of nose-poke responses was stable. The number of nose-pokes executed, and the rewards obtained were recorded using the LabStat Standard Edition software. In the final 3 days, mice with fewer than 10 active nose-poke responses were excluded from further experiments.

### Immunohistochemistry

Arc is vital to the formation of memory and its expression changes dynamically during the learning process. A previous study has shown that the expression of Arc increases over a period of 30 min to 2 h upon the increase in network activity, exposure to new environments, or detailed study programs (Wall et al., 2018). To assess the expression of Arc during instrumental learning, the mice were sacrificed 90 min after the time they were placed in the box. Mice in the training group were required to undergo the FR1 program, while mice in the control group were just placed in the operation box, without the program being performed. Mice were anesthetized with pentobarbital (50 mg/kg, i.p.) and the left ventricle was perfused with 0.9% normal saline followed by fixation with 4% paraformaldehyde (PFA). Brains were harvested and placed in 4% PFA overnight. Sections of brain tissue (40-μm thick) were prepared with a concussion microtome, and then placed in 0.01 M PBS for immunohistochemistry. The floating slices containing striatum were rinsed in PBS three times. The sections were then incubated overnight at 4°C with polyclonal antibody of rabbit-anti-Arc (SYSY, 156002) diluted with 0.25% Triton X-100 (v/v) and 3% normal donkey serum (v/v) (1:1,000). After washing in PBS at least three times, the sections were transferred into biotinylated goat-anti-rabbit antibody (Vector laboratories, BA1000) that was diluted with 0.25% Triton X-100 (v/v) (1:1,000) at 37°C for 1 h. After repeated washing in PBS, the sections were incubated with Alexa Fluor 546 (1:1,000; Invitrogen) for 2 h at room temperature. Finally, the sections were rinsed in 0.01 M PBS.

### Imaging Analysis

A train of brain sections containing striatum was imaged under a confocal microscope equipped with a 20× objective lens (Olympus, Japan). To facilitate a comparison between the groups, all fluorescence images in the experiment were assessed using the same laser and scan settings. The photoshop software counting tool was applied to manually count the Arc immunoreactive cells in the DMS and DLS. Arc-positive cells were marked and calculated by different investigators, and the average values were used for statistical analysis. Arc-positive cells in brain sections of striatum were calculated in an area about + 1.10 to −0.10 mm from the anterior to the posterior direction (Gong et al., 2020; Figure 2B).

### Statistical Analysis

All data are expressed as mean ± standard error of the mean (SEM). The mean scores for the behavioral and immunohistochemical experiments were subjected to multivariate analysis of variance. Multiple comparisons were performed using Tukey *post hoc* analysis. Data management and analysis were performed using GraphPad Prism 8 software. *P* values < 0.05 were considered indicative of statistical significance.

## Results

### Acquisition of FR1 Response for Sucrose

**FIGURE 1:**
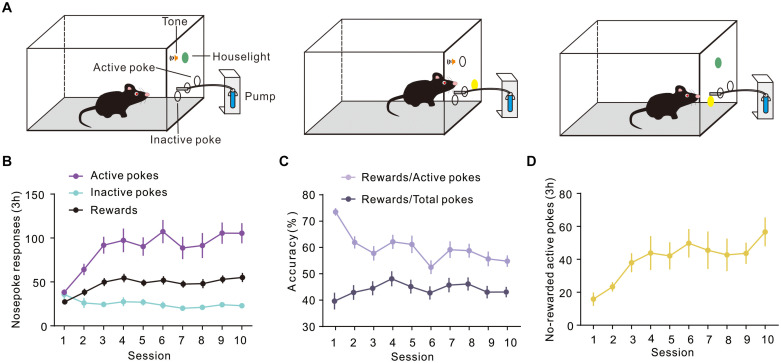
Performance of sucrose self-administration behavior. **(A)** Schematic illustration of sucrose self-administration. **(B)** Under the fixed ratio 1 (FR1) program, active and inactive nose pokes in response to 10% sucrose were calculated during training (*n* = 20). **(C)** Curve showing the proportion of the number of rewards and the number of pokes. **(D)** The number of No-rewarded active pokes in 3 h.

To evaluate the expression of Arc during different periods of instrumental learning, mice were self-administered sucrose using the conventional training protocol described in a previous report (Mahler and Aston-Jones, 2012). In our study, mice were trained in sucrose SA for 3 h a day, for 10 consecutive days in operant conditioning boxes. The design of the box is shown in Figure 1A. Sucrose flows out when the mouse nose touches the active port, at which point the house light was turned on and a sound was activated to indicate that a reward was available. On the contrary, there were no programmed consequences upon touching another (inactive) nose-poke port. Figure 1B shows the total number of active and inactive nose pokes during the training process of the FR1 response to the sucrose solution. Optional increase in nose poking on the active hole reached an asymptote in 10 sessions. The number of rewards also increased on consecutive days, reaching a plateau on the third day, which was maintained until the 10th day. It is worth noting that the rate of rewards and the ratio of active pokes declined. The number of pokes without rewards increased, which means that the correct rate of poke decreased during training (Figures 1C,D).

### Regional Specificity of Arc Protein Expression in the Dorsomedial and Dorsolateral Striatum During Instrumental Learning

**FIGURE 2:**
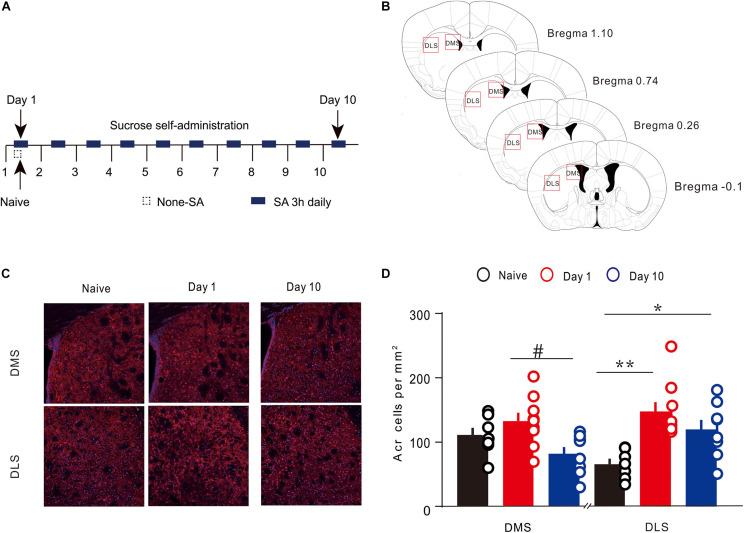
Expression of Arc in the specific regions of the dorsal striatum during sucrose self-administration. **(A)** Self-administration of sucrose solution in mice (*n* = 20). Arrows represent the time of sampling. **(B)** George and Franklin’s (2001) coronal section map with Bregma coordinates. Reference modified from Gong et al. (2020), quantitative explanation of Arc in the brain regions. **(C)** Representative images of the DMS and DLS showing Arc expression in different groups on day 1 and day 10. Scale bar = 300 μm. **(D)** The density of Arc^+^ cells in the DMS and DLS on day 1 (*n* = 9), day 10 (*n* = 8), and in the naive group (*n* = 7) mice. *P < 0.05, **P < 0.01 versus naive group by one-way ANOVA. #*P* < 0.05 versus day 10 in the DMS by Tukey *post hoc* tests. Data are expressed as mean ± SEM. cc, corpus callosum DMS, dorsomedial striatum; LV, lateral ventricle; IHC, immunohistochemistry; DLS, dorsolateral striatum.

To detect the Arc expression in the different regions of the dorsal striatum, during various periods of instrumental learning, all brain tissues of mice were obtained on the first and 10th day of training (Figures 2A,B). The expression of Arc in the DMS changed dynamically during the 10-day sucrose SA process [one-way ANOVA, *F*(2,21) = 4.724, *P* < 0.05]. On the first day, there was no significant increase in the number of Arc-positive cells (Figures 2C,D), while there was an obvious difference of Arc-positive cells between day 10 and day 1 (Figures 2C,D). Results of the *post hoc* Tukey test showed that the density of Arc-positive cells on day 1 was significantly higher (132.67 ± 13.14 cells/mm^2^, 8 *n* = 9; vs. 81.36 ± 11.07, *n* = 8 *P* < 0.05) than that on day 10. The expression of Arc in the DLS also showed a dynamic change during the 10-day sucrose SA process [one-way ANOVA, *F*(2,21) = 8.884, *P* < 0.01]. Results of the *post hoc* Tukey test showed that the density of Arc-positive cells in the DLS was significantly higher on day 1 (147.39 ± 14.71, *n* = 9 vs. 65.56 ± 8.49 cells/mm^2^, *n* = 7; *P* < 0.01) and on day 10 (119.25 ± 15.19, *n* = 8 vs. 65.56 ± 8.49 cells/mm^2^, *n* = 7; *P* < 0.05) as compared to that in the naive group. In addition, the expression of Arc in DLS did not differ between day 1 and day 10. These results indicate that Arc showed area-specific expression in the striatum during the instrumental learning process.

### Correlation Between the Number of Rewards and the Density of Arc^+^ Cells

**FIGURE 3:**
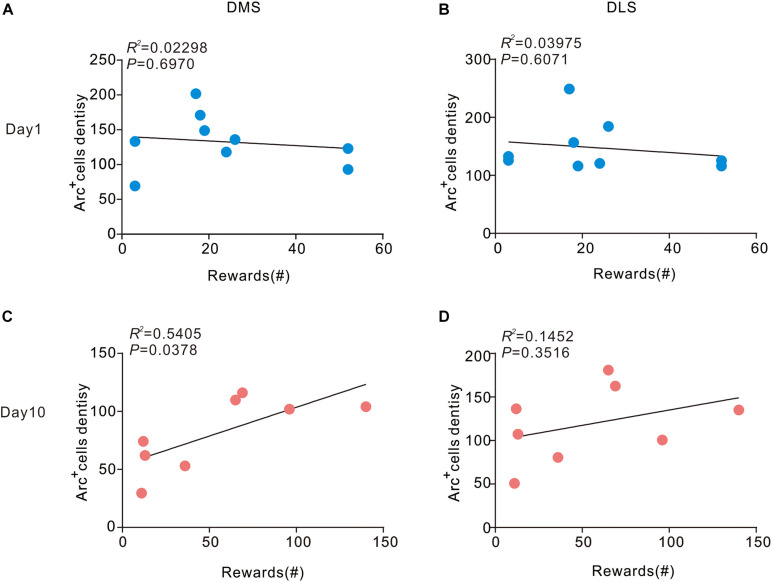
Correlation between Arc expression and reward times in the DMS and DLS. **(A,B)** Arc expression during instrumental learning did not correlate with sucrose self-administration behavior on day 1 (*n* = 9). **(C)** Day 10, the density of Arc positive cells correlated with the magnitude of the number of rewards in the DMS (*P* < 0.05, *R*^2^ = 0.5405) Pearson’s product-moment correlation coefficients. **(D)** In the DLS, Arc expression did not correlate with the number of rewards on day 10. *n* = 8 mice for the day 10 group.

Next, we sought to assess the correlation between density of Arc-positive cells in the dorsal striatum and behavior. We conducted a correlation analysis to determine the relationship of current behavioral data with Arc expression in the striatum during different stages of sucrose SA. On day 1, no pronounced correlation was observed in DMS (*R*^2^ = 0.02298, *P* > 0.05) and DLS (*R*^2^ = 0.03975, *P* > 0.05) (Figure 3). However, in the DMS, the number of rewards showed a significant positive correlation with the density of Arc^+^ cells on day 10 (*R*^2^ = 0.3115, *P* > 0.05). There was also no apparent association between the number of rewards and the density of Arc^+^ cells in the DLS (*R*^2^ = 0.1452, *P* > 0.05) on day 10.

## Discussion

In this study, we investigated instrumental learning using sucrose SA in which thirsty animals can obtain sucrose water by poking the active port. The results showed region-specific expression of Arc in the dorsal striatum during the formation and stabilization stage of SA instrumental learning. Arc expression in the DMS was increased during the early stage of instrumental learning and reduced to the basic levels when the procedural operation was automated; in contrast, Arc expression in the DLS was increased both in the early stage and the later stage.

A wide body of evidence supports the essential role of the basal ganglia circuit in instrumental learning and operation (Groenewegen, 2003). However, these two functions are generally considered to be distinct and independent. Instrumental learning mainly depends on glutamatergic projections from the frontal cortex and limbic lobe to the dorsal striatum, while the execution of instrumental learning tasks is primarily dependent on the dorsal striatal output pathways (Kreitzer and Malenka, 2008). Previous studies have shown the involvement of dorsal striatum in several kinds of learning (Graybiel and Grafton, 2015); however, the precise dynamic role of the DLS and DMS in SA instrumental learning is not well characterized (Devan et al., 2011; Kupferschmidt et al., 2017). From a neuroanatomical perspective, the DMS and DLS are two distinct regions which receive different projections from the cortex and the midbrain dopaminergic nucleus (Hintiryan et al., 2016). The DMS receives afferents from the cognition-related cortex, such as the prefrontal cortex and the associative cortex, while the DLS receives afferents from the motor execution cortex (Voorn et al., 2004; Hawes et al., 2015).

The sucrose SA can be roughly divided into two stages: (1) the early learning/training stage (first 3 days) during which the mouse learns how to operate the equipment motivated by the reward (sucrose); and (2) the later stage (4 to 10 days) when the mouse has already learnt and transferred it into a proficient skill and may perform it habitually. During the early stage of training, the mouse initially explores the environment aimlessly; it typically pokes the active side and inactive side randomly until it obtains the reward (sucrose) after an active nose poke, which is followed by a light and tone; subsequently, it tends to poke more purposively to obtain sucrose. As shown in Figure 1, nose pokes of the two sides are almost equal at first (session 1); however, the active pokes increased very soon after (especially the first three sessions), with a simultaneous gradual decrease in the inactive pokes. The prefrontal cortex and DMS are mainly involved in this process of cognition; consistently, DMS was preferentially activated in the early stage with an increase in Arc expression. Previous studies have shown that the DMS plays an important part in the early acquisition of dynamic foraging, motor skill learning (Sheng et al., 2019), habitual drug seeking (Corbit et al., 2012), and instrumental learning (Vicente et al., 2016; Peak et al., 2019). Our results are consistent with the results of previous studies.

With the progression of training in the later stage (4–10 days), mice had already learnt and transferred the learning into a proficient skill. The reduced involvement and dependence of DMS led to downregulation of Arc in this stage. The characteristics of Arc expression showed that the DLS is activated during both the early and later stages of instrumental learning. Earlier studies have shown the participation of DLS in the consolidation of skilled actions in operant tasks, habitual drug-seeking, and motor skill learning (Kimchi and Laubach, 2009; Thorn et al., 2010; O’Hare et al., 2016). The results of Arc expression in our study are consistent with those of previous studies, indicating that Arc expression is indispensable for the acquisition and consolidation of instrumental learning.

Immediate early genes, including Arc/Arg3.1 and c-Fos, are a group of genes that are expressed dynamically and rapidly in brain regions that are closely related to the formation of memory and learning procedures (Ramírez-Amaya et al., 2005). The protein products of immediate early genes have been regarded as biomarkers of neurons that activate or exhibit plasticity changes. In a previous study, expression of c-Fos was shown to increase in the dorsal part of the striatum during motor learning; however, no dynamic change was observed among the different phases of learning (Bureau et al., 2010). This suggests that c-Fos may not be an appropriate biomarker for representing dynamic changes of plasticity between the different motor learning stages (Yin et al., 2009). However, a previous study documented dynamic changes in the expression of Arc in the dorsal striatum during the learning phase of a touchscreen task (Bergstrom et al., 2018). At the molecular level, Arc is functionally involved in the maintenance of homeostatic synaptic plasticity by accelerating endocytosis of AMPA-type glutamate receptors (Tzingounis and Nicoll, 2006; Jakkamsetti et al., 2013). Our study indicates region-specific dynamic changes in Arc expression in the dorsal striatum during SA learning. Therefore, Arc is an eligible biomarker for neurons that undergo plasticity transformation during different phases of learning. With a decrease of Arc expression in the DMS while showing an obviously positive correlation with the number of rewards received in the later stages, our study shows a dissociation between Arc expression in the DMS and learning stages in the instrumental learning of sucrose SA.

In summary, our study demonstrates regional and temporal variations of neural plasticity in DMS during different phases of sucrose SA instrumental learning through Arc expression.

## Data Availability Statement

The original contributions presented in the study are included in the article/supplementary material, further inquiries can be directed to the corresponding authors.

## Ethics Statement

The animal study was reviewed and approved by the Ethics Committee of Medical College of Shanghai Jiao Tong University.

## Author Contributions

YL, W-KG, G-QX, and T-FY designed the experiment. XL, J-WZ, QD, and CW collected the data. XL, J-WZ, QD, Y-CG, W-QL, and DW analyzed the data. XL, J-WZ, and QD wrote the manuscript. All authors contributed to the article and approved the submitted version.

## Conflict of Interest

The authors declare that the research was conducted in the absence of any commercial or financial relationships that could be construed as a potential conflict of interest.
